# Sentiment Analysis of Patient- and Family-Related Sepsis Events: Exploratory Study

**DOI:** 10.2196/51720

**Published:** 2024-04-01

**Authors:** Mabel Ntiamoah, Teenu Xavier, Joshua Lambert

**Affiliations:** 1 University of Cincinnati Cincinnati, OH United States

**Keywords:** families, patients, sentiment analysis, sepsis

## Abstract

**Background:**

Despite the life-threatening nature of sepsis, little is known about the emotional experiences of patients and their families during sepsis events. We conducted a sentiment analysis pertaining to sepsis incidents involving patients and families, leveraging textual data retrieved from a publicly available blog post disseminated by the Centers for Disease Control and Prevention (CDC).

**Objective:**

This investigation involved a sentiment analysis of patient- and family-related sepsis events, leveraging text responses sourced from a publicly accessible blog post disseminated by the CDC. Driven by the imperative to elucidate the emotional dynamics encountered by patients and their families throughout sepsis incidents, the overarching aims centered on elucidating the emotional ramifications of sepsis on both patients and their families and discerning potential avenues for enhancing the quality of sepsis care.

**Methods:**

The research used a cross-sectional data mining methodology to investigate the sentiments and emotional aspects linked to sepsis, using a data set sourced from the CDC, which encompasses 170 responses from both patients and caregivers, spanning the period between September 2014 and September 2020. This investigation used the National Research Council Canada Emotion Lexicon for sentiment analysis, coupled with a combination of manual and automated techniques to extract salient features from textual responses. The study used negative binomial least absolute shrinkage and selection operator regressions to ascertain significant textual features that correlated with specific emotional states. Moreover, the visualization of Plutchik’s Wheel of Emotions facilitated the discernment of prevailing emotions within the data set.

**Results:**

The results showed that patients and their families experienced a range of emotions during sepsis events, including fear, anxiety, sadness, and gratitude. Our analyses revealed an estimated incidence rate ratio (IRR) of 1.35 for fear-related words and a 1.51 IRR for sadness-related words when mentioning “hospital” in sepsis-related experiences. Similarly, mentions of “intensive care unit” were associated with an average occurrence of 12.3 fear-related words and 10.8 sadness-related words. Surviving patients’ experiences had an estimated 1.15 IRR for joy-related words, contrasting with discussions around organ failure, which were associated with multiple negative emotions including disgust, anger, fear, and sadness. Furthermore, mentions of “death” were linked to more fear and anger words but fewer joy-related words. Conversely, longer timelines in sepsis events were associated with more joy-related words and fewer fear-related words, potentially indicating improved emotional adaptation over time.

**Conclusions:**

The study’s outcomes underscore the imperative for health care providers to integrate emotional support alongside medical interventions for patients and families affected by sepsis, emphasizing the emotional toll incurred and highlighting the necessity of acknowledgment and resolution, advocating for the use of sentiment analysis as a means to tailor personalized emotional aid, and thereby potentially augmenting both patient and family welfare and overall outcomes.

## Introduction

Sepsis is a life-threatening medical emergency that affects millions of people worldwide each year. It is estimated that sepsis affects over 30 million people worldwide annually, resulting in over 6 million deaths each year [1], with a substantial economic burden and long-term morbidity among survivors [2]. It is characterized by a dysregulated immune response to an infection, leading to organ dysfunction and, in severe cases, mortality. Despite advances in sepsis care, the high mortality rate underscores the need for a comprehensive understanding of the patient’s experience.

The concept of patient-centered care has gained recognition in health care, highlighting the importance of incorporating patient perspectives, needs, and preferences into the care delivery process [3]. Within the context of sepsis, understanding the emotional experiences of patients and their families during sepsis events is crucial for providing holistic and patient-centered care. Although there is a lack of extensive research on the emotional experiences of patients and their families, specifically during sepsis events, studies conducted in related fields highlight the crucial role of emotional support and its impact on patient outcomes [4]. For instance, in critical care settings, emotional distress and psychological well-being have been shown to significantly influence patient recovery and quality of life [5,6]. Similarly, in chronic illness contexts, emotional support has been linked to improved patient coping, treatment adherence, and overall well-being [7].

In sepsis care, it is important to recognize and address the emotional needs of both patients and their families [8]. This approach contributes to a more comprehensive and patient-centered method of care [9]. Numerous studies indicate that providing emotional support during critical illness can alleviate anxiety, reduce psychological distress, and improve overall satisfaction with care for patients and their families [10,11].

To gain a deeper understanding of the emotional experiences of patients and their families, researchers have turned to sentiment analysis as a valuable technique [12]. Sentiment analysis has gained prominence in recent years as a powerful tool for comprehending patients and health care workers’ experiences, opinions, and attitudes toward health care [13,14]. Sentiment analysis is a computational approach that analyzes the emotional tone or sentiment expressed in text data [15]. By applying sentiment analysis to patient and family feedback related to sepsis events, health care providers can better understand the emotional impact of sepsis on patients and their families and identify areas for improvement in sepsis care. Furthermore, analyzing patient and family feedback can aid health care providers in comprehending the patient’s sepsis experience and developing strategies to enhance sepsis care [16].

This study aims to perform a sentiment analysis on the experiences of patients and their families during sepsis events gathered from 174 narratives, with the goal of comprehending the emotional toll of sepsis and pinpointing opportunities for enhancing sepsis care. Through the examination of feedback from patients and their families, the study seeks to enrich the existing literature on sepsis care. The insights gained from this study are poised to equip health care providers with insights that could lead to better management of patient and family emotional needs.

## Methods

### Overview

Sepsis-related patient and caregiver text responses were obtained from a public data set provided by the Centers for Disease Control and Prevention (CDC), originating from their Division of Healthcare Quality Promotion Public Inquiries Team. The CDC data set, collected between September 16, 2014, and September 19, 2020, comprised 174 comments. In a 2014 blog post titled *A Family’s Perspective - “The Brutality of Sepsis will Haunt Us for the Rest of Our Lives”* [16], author Franchot Karl describes his 84-year-old grandmother’s death from sepsis and offers advice for those yet to be affected by the disease. The comments were reader descriptions of their personal experiences related to sepsis and a direct response to the 2014 blog post.

These reader comments were subsequently downloaded and exported to Excel (Microsoft Corporation) and analyzed using JMP Pro (version 16; SAS Institute), R (version 4; R Core Team), and Python (version 3.9; Python Software Foundation).

A total of 4 responses were removed due to missing information or spam-related comments. The remaining 170 responses submitted by patients and caregivers were analyzed using the National Research Council Canada (NRC) Emotion Lexicon, which produced 8 distinct emotional sentiment scores [17]. The lexicon emphasizes unigrams, with each word assigned ratings based on its positive or negative sentiment and potential association with emotions, including anger, fear, anticipation, trust, surprise, sadness, joy, and disgust. The scoring method involved tabulating the number of words in each response that received at least 1 of the 8 sentiment scores and categorizing the number of words in each response that expressed each emotion. This generated 8 count variables, which served as the study’s outcome variables.

The research team implemented a rigorous, multistep manual and automated process to extract diverse features from the text responses. This entailed a comprehensive analysis of each response to identify recurring or predetermined features, such as patient age, respondent relationship to the patient, inferred sex of the patient and caregiver, patient survival, and sepsis-related conditions like severe sepsis and septic shock. Additionally, indicator variables (1 or 0) were developed for frequently occurring words, such as “sepsis,” “hospital,” and “doctor.” The team compiled a list of recurrent sepsis-related terms by examining the responses, which were then used to create indicator variables—assigned a value of “1” if present in the text and “0” if not present in the text. In instances of coding discrepancies, the team reviewed the responses and deliberated to reach a consensus. These indicators played a crucial role in tackling elements of our research questions, particularly in identifying dominant themes or subjects within the sepsis events involving patients and their families. Temporal references, such as hours, months, days, and years, were classified as timelines, which were further categorized into short and long timelines. Short timelines could only include terms such as “suddenly,” “days,” “hours,” “immediately,” “quickly,” “seconds,” and “currently,” whereas all other timelines were deemed long timelines. Due to concerns regarding reliability and substantial missing data, textual information such as age and the sex of the caregiver and patient was excluded from the analysis. However, a separate bivariate analysis was conducted to explore the potential influence of these variables on the 8 NRC emotions.

A total of 8 negative binomial least absolute shrinkage and selection operator (LASSO) regressions [18] were used to identify patient and caregiver text response features associated with the count of anger, anticipation, disgust, fear, joy, sadness, surprise, and trust sentiments in the responses. A negative binomial was chosen a priori over Poisson regression as it was believed the outcomes would likely be over dispersed. Upon inspection, the data were not zero-inflated, so zero-inflated models were not considered. Model selection was performed by selecting the model that had the smallest Akaike information criterion corrected [19].

To visualize Plutchik’s [20] Wheel of Emotions, we used the *PyPlutchik* (Alfonso Semeraro) Python package [21]. This package offers functionality to generate visual representations of the Wheel of Emotions proposed by Plutchik [20]. To determine the dominant emotion within the wheel, we selected the emotion with the highest frequency and assigned it a score of 1. We then calculated the scores for the remaining emotions based on their ratio to the dominant emotion. As a result, emotions within the wheel are scored on a scale from 0 to 1, with 1 being the emotion that occurred most frequently.

### Ethical Considerations

This study was conducted in accordance with ethical standards regarding research involving nonhuman subjects. The ethics committee of the University of Cincinnati granted approval on May 5, 2023, for the study (2023-0396). The privacy of participants' personal information was rigorously protected, securely stored, and only accessible by the study team.

## Results

Variables with reliability issues, missing values, or chosen to not be included in the analysis were first checked bivariately with the outcome variables. None of these variables were related bivariately to the 8 outcomes and were therefore not included in any further analysis.

Table 1 presents a numerical summary of the average values for the 8 NRC emotions, as categorized by extracted text features. The estimates are obtained column-wise, with larger numbers signifying an increase in the number of words associated with a particular emotion, while a smaller number denotes a decrease in the number of words related to that emotion. For instance, responses containing “medical” terminology (medical professionals, medical issues, medical field, medical records, and medical history) exhibited an average of 7.7 anticipation words. An increase in anticipation words could be considered unfavorable in this context. Conversely, joy and trust have inverse scales, as a greater presence of joy or trust emotions signifies improvement compared to fewer instances of joy or trust. When responses included “medical” terminology, an average of 12.4 trust words were observed. From these 2 results, we can see that, consequently, the “medical” terminology indicator is associated with both heightened anticipation and increased trust. Owing to the extensive results presented in Table 1, further insights are elaborated upon in the discussion section and are also available for the reader to examine independently. Due to the large number of results in Table 1, the discussion section has an overview of the results that the authors found interesting or notable. The readers are encouraged to review Table 1, as it may present other, undiscussed findings.

**Table 1:** Average of the 8 National Research Council Canada emotions by gathered text features.

Columns by categories			Number, n		Anger		Anticipation		Disgust		Fear		Joy		Sadness		Surprise		Trust
Alive			55		3.8		4.9		3.4		11.6		3.4		9.9		2.0		6.8
Sepsis indicator			125		4.0		4.8		3.6		11.5		2.9		10.5		2.3		7.0
Hospital indicator			111		4.4		5.3		4.3		12.8		3.1		11.8		2.6		8.4
Doctor indicator			67		5.3		6.5		5.2		14.5		3.8		13.5		3.2		10.0
Pain indicator			46		5.3		6.0		4.5		15.4		3.7		14.2		2.5		8.5
Surgery indicator			40		6.2		6.1		5.8		16.6		3.3		15.4		3.1		8.9
Infection indicator			48		5.2		5.4		4.7		15.0		3.3		12.3		2.9		8.4
Heart indicator			42		6.3		7.1		6.3		16.1		4.1		14.4		3.3		10.4
Septic indicator			49		5.7		4.8		5.7		12.8		2.7		11.3		3.2		7.0
Shock indicator			53		5.1		4.3		4.6		11.0		2.5		9.6		3.1		6.2
ED^a^ indicator			31		5.5		6.8		6.2		15.9		3.7		14.2		3.5		10.3
Medical indicator			34		6.5		7.7		5.7		17.3		3.7		15.1		3.3		12.4
Antibiotics indicator			36		5.1		6.8		5.3		16.7		3.6		14.3		3.3		10.6
ICU^b^ indicator			32		4.5		5.0		4.9		12.3		3.3		10.8		2.9		7.3
Symptoms indicator			29		5.3		5.6		4.3		14.8		3.6		14.0		2.7		9.3
Fever indicator			21		4.3		4.9		4.2		15.5		2.9		12.9		2.3		8.6
Failure indicator			20		6.7		7.5		8.7		16.8		3.9		15.7		3.9		10.9
Death indicator			72		4.3		4.4		4.0		11.0		2.6		10.1		2.6		7.0
Long timeline			116		4.2		5.1		4.3		11.8		3.2		11.0		2.6		7.8
Short timeline			34		3.4		2.9		2.5		8.3		1.5		6.8		1.4		4.2
**Responder**																			
	Child	88		3.7		4.1		3.4		9.9		2.5		9.3		1.7		6.2	
	Patient	27		5.6		6.1		4.6		14.9		4.2		12.7		2.9		8.6	
	Spouse	26		2.7		3.8		2.8		7.8		2.0		7.4		2.3		6.0	
	Parent	7		2.6		3.3		4.3		7.7		2.6		7.1		2.4		5.0	
	Other	22		3.1		3.5		3.2		9.9		2.3		8.9		2.5		6.3	

^a^ED: emergency department.

^b^ICU: intensive care unit.

Table 2 presents a numerical summary of the 8 NRC emotions negative binomial LASSO regression model estimates. The contents of these 8 multivariable models are presented in Table 2 column-wise, where variables that were included in the model have presented a numerical estimated regression coefficient, while variables that were not chosen by the LASSO procedure have a “–” in their cell. The estimates are obtained column-wise, with larger numbers signifying an increase in the estimated rate of words associated with a particular emotion, while smaller numbers signify a decrease in the estimated rate of words related to that emotion. For example, the model for NRC anger suggested that respondents who used the word “medical” were estimated to have 1.75 times the number of anger-related words than those respondents who did not use the word “medical.” As with Table 1, there are many results in Table 2, and we discuss these further in context within the discussion section. The readers are encouraged to review Table 2, as it may present other, undiscussed findings.

**Table 2:** Negative binomial least absolute shrinkage and selection operator regression parameter estimates.

Columns by categories			Anger		Anticipation		Disgust		Fear		Joy		Sadness	Surprise		Trust
Alive			—^a^		1.09		—		—		1.15		—	—		—
Sepsis indicator			1.19		1.19		1.14		1.39		1.07		1.33	1.24		—
Hospital indicator			1.01		1.25		—		1.35		1.12		1.51	1.08		1.77
Doctor indicator			1.30		1.39		1.58		1.29		1.34		1.29	1.61		1.42
Pain indicator			1.32		1.36		1.22		1.44		1.31		1.50	—		1.25
Surgery indicator			1.13		—		1.13		1.20		—		1.26	1.03		—
Infection indicator			—		—		—		1.14		—		—	1.10		—
Heart indicator			1.32		1.49		1.09		1.26		1.42		1.23	1.02		1.40
Septic indicator			1.33		—		1.74		1.12		—		1.11	1.14		—
Shock indicator			1.25		—		—		1.03		—		—	1.82		—
ED^b^ indicator			1.12		1.14		1.32		1.11		1.07		1.14	1.22		1.11
Medical indicator			1.75		1.55		1.55		1.47		1.11		1.39	1.28		1.64
Antibiotics indicator			—		1.03		—		1.12		—		1.02	1.13		1.06
ICU^c^ indicator			—		—		—		—		—		—	—		—
Symptoms indicator			—		—		0.77		—		—		—	—		1.08
Fever indicator			—		—		—		1.26		—		1.16	—		1.11
Failure indicator			1.49		1.26		2.02		1.29		—		1.28	1.49		1.14
Death indicator			1.09		—		—		1.03		0.90		—	—		—
Any timeline			1.30		—		1.47		1.22		—		1.03	1.17		—
Long timeline			1.05		1.32		1.56		1.03		1.62		1.20	1.65		1.25
Short timeline			—		—		—		—		—		—	—		—
**Responder**																
	Child	—		—		—		—		—		—		0.68	—	
	Patient	1.57		1.15		1.30		1.23		1.17		1.12		1.05	1.12	
	Spouse	—		—		0.98		—		0.96		—		—	—	
	Parent	0.89		—		1.12		—		—		—		0.89	—	
	Other	0.95		0.91		—		—		—		—		—	—	

^a^Not available.

^b^ED: emergency department.

^c^ICU: intensive care unit.

Table S1 in Multimedia Appendix 1 presents various model fit details (number of parameters, Bayesian information criterion, corrected Akaike information criterion, and dispersion) for the 8 negative binomial LASSO regression models. These fit details are provided for transparency and reproducibility. Table S2 in Multimedia Appendix 1 presents a correlation matrix of the 8 NRC emotion outcome. Sadness and fear (*r*=0.9405) had the strongest correlation, while anger and joy (*r*=0.5755) had the weakest correlation of all possible emotional pairs.

**Figure 1:**
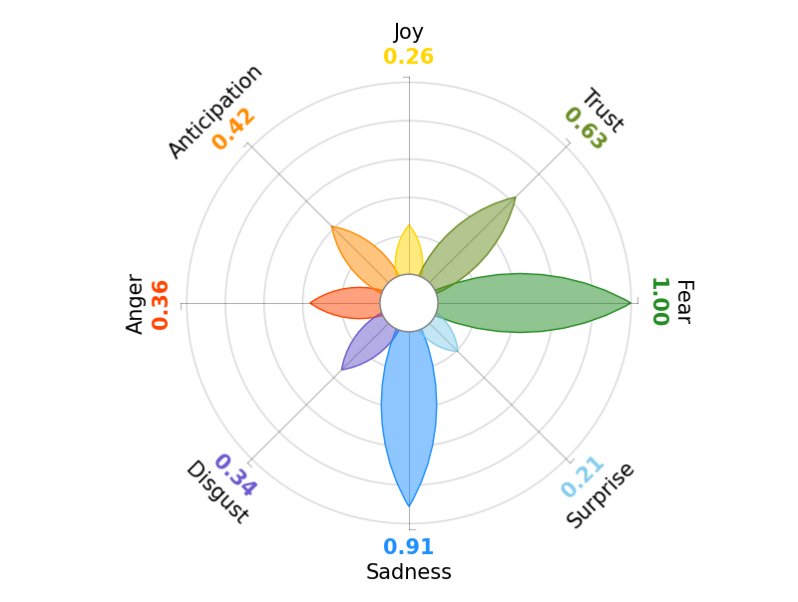
Plutchik Wheel of Emotions of all sepsis-related responses.

Figure 1 display the Wheel of Emotions [13] for the sample overall.

## Discussion

### Overview

This study examined the emotional responses of patients and caregivers to sepsis-related events using sentiment analysis. The analyzed text responses from 170 patients, caregivers, children, spouses, and others showed that there were numerous text features that indicated elevated emotional patterns and trends. These patterns and trends are discussed in the following subsections.

#### Medical, Hospital, Intensive Care Unit, Sepsis, and Emergency Department Indicators

Our analysis revealed that there was a higher occurrence of fear- and sadness-related words when hospital, sepsis, or emergency department (ED) were mentioned in sepsis-related experiences (Table 2). For example, when the word “hospital” is mentioned, there is a 1.35 times higher frequency of fear-related words and a 1.51 times higher frequency of sadness-related words. These findings underscore the profound emotional impact of sepsis, which is characterized by its critical nature and the uncertainty surrounding its prognosis, leading to heightened emotional distress. Moreover, the traumatic aspects of sepsis, including its sudden onset, severe symptoms, near-death experience, and the urgent need for immediate medical intervention and life support, can cause psychological trauma and further contribute to intensified feelings of fear and sadness [22-24]. The admission of a patient to the hospital or ED can be particularly shocking for patients, families, and friends, especially when the illness was unexpected. The constant highs and lows can be emotionally draining when the future is uncertain, with worries about losing loved ones or coping with disabilities from sepsis [24]. Our findings are consistent with the findings of Apitzsch et al [22] and Gallop et al [24] who qualitatively explored the mental impact of surviving sepsis and discovered that survivors often harbor a great sense of fear of experiencing sepsis again and becoming critically ill again.

The “medical” indicator was associated with heightened anticipation and anger as well as increased trust. Patient experiences may trigger heightened anticipation or concern due to the seriousness of the medical matters discussed. This association is reflected in the increased usage of words associated with anticipation within these contexts. Simultaneously, the concurrent rise in expressions of trust when “medical” terms are used implies that, despite heightened anticipation or worry, respondents also demonstrate a level of trust or reliance on medical professionals or care within these conversations. Moreover, the discovery that individuals using the term “medical” tend to express more words associated with anger suggests potential frustration or discontent during discussions involving medical elements. Recognizing these connections holds significance for health care professionals, indicating the necessity for improved communication strategies to address patient and family apprehensions. This emphasizes the importance of conveying medical terminologies in a manner that minimizes negative emotional reactions, ultimately enhancing patient and family experiences in navigating such discussions.

The results of our analysis indicated an increase in the average occurrence of words related to fear and sadness when the term “ICU” was mentioned. Specifically, fear-related words had an average occurrence of 12.3, while sadness-related words had an average occurrence of 10.8 (Table 1). These findings align with a study conducted by Kang et al [25], who performed sentiment analysis on responses from intensive care unit (ICU) survivors and reported the highest scores for sadness and fear. This consistency in findings suggests that the mention of the ICU in sepsis-related experiences elicits heightened emotional expressions of fear and sadness, reflecting the emotional impact of the ICU environment and the experiences associated with a critical illness. However, interestingly, ICU was not included in the 8 regression models, suggesting that other factors, possibly related to ICU exposure, better explain the variation noted by the word “ICU.”

#### Life, Failure, Death, and Shock

Our findings suggest that if the outcome of the patients were alive, the responses had 1.15 times more joy-related words. Surviving patients and relatives often experience relief and joy after recovering from a serious illness such as sepsis. The recovery of a patient can lead to an overall increase in the positive language used when discussing the patient’s outcomes or experiences. This was also noted by Papathanassoglou and Patiraki [26], who investigated the long-term effects of critical illness on survivors and found that participants frequently highlighted emotions of personal transformation, joy, and a newfound appreciation for the wonders of life.

Sepsis is a complication of infection that often leads to organ failure, including the heart, kidneys, respiratory organs, and liver [27]. The results of this study reveal a noteworthy trend: when respondents discuss failure in relation to organ failures, such as kidney failure, liver failure, or heart failure, their responses tend to contain a higher frequency of words associated with disgust, anger, fear, sadness, and surprise. This observation can be attributed to the fact that organ failure is a severe and potentially life-threatening condition that profoundly affects both patients and their families. They experience psychological burdens due to feelings of indefinite care over time, and constant uncertainty, and worry about deteriorating health and death [28].

Given the significant emotional impact of organ failure, it is crucial for health care providers to recognize this and offer appropriate emotional support and resources. By acknowledging and addressing the emotional challenges faced by patients and their families in addition to the physical ones, health care professionals can enhance the overall well-being and coping mechanisms of those affected by organ failure [28]. It is imperative to shift the perspective and no longer view the family as merely a resource for patient care but instead integrate them into the health care process, valuing their input and involving them in decision-making.

Our findings suggest that the mention of the word “death” was associated with more fear- and anger-related words and fewer joy-related words. The mention of death or the patient’s death due to sepsis in responses can signal a significant and often tragic event that has occurred, which can contribute to the overall emotional tone of the language used. The mention of death can be associated with feelings of loss, helplessness, and regret, which can diminish the experience of joy. The finding that responses containing the word “death” had more fear-related words suggests that the concept of death can evoke fear in individuals. The relatives of the patients might be terrified because of the unexpected death of patients due to sepsis and its sudden onset, which might provoke feelings of anger as they navigate through the grieving process [29]. The unexpected death of a loved one is widely recognized as one of the most profoundly traumatic experiences in an individual’s life [30]. In the context of sepsis, the fear experienced by relatives following the death of a patient can stem from various factors. These may include the fear of losing someone dear to them, the fear of not understanding the exact cause of death, and the fear of the unknown. The emotional impact of such circumstances can be immense, underscoring the importance of providing support to bereaved relatives during the grieving process.

The results indicate that if the word “shock” is mentioned in a response, there is an increased occurrence of words associated with surprise. Shock developed due to sepsis can be a serious and potentially life-threatening condition and is often the most common cause of death [31] that can lead to a range of physical and emotional responses. The experience of shock may be unexpected and sudden, leading to a heightened emotional response that includes surprise. Additionally, shock can be categorized as a word that depicts surprise, and it is possible that the sentiment analysis might have considered shock as a factor of surprise rather than its specific context related to sepsis. It is also possible that when counting the number of NRC surprise words, “shock” was counted as a surprise word, as another definition of “shock” is a sudden upsetting or surprising event or experience. This additional count could have led to increases in this count variable, and thus showing an increase in our tables.

#### Timelines

Long timelines, as compared to a short or quick timeline, related to sepsis and sepsis-related events had more joy-related words and fewer fear-related words. Patients who make progress toward recovery and achieve improvements in their health may experience joy and satisfaction as they reach milestones and see improvements in their quality of life. Long timelines may allow individuals to gain a broader perspective and distance themselves from the initial stress and fear of the illness, enabling them to concentrate on more optimistic aspects such as recovery and healing. That may be the reason for the reduction in the frequency of fear-related words in their responses. Additionally, as time passes, individuals and family members may have had more opportunities to process their experiences and emotions related to sepsis, potentially leading to a greater sense of acceptance, peace, and gratitude. These findings are consistent with a review conducted by Paul and Rattray [32], which examined the short- and long-term impact of critical illness on relatives. The review reported that emotional distress among relatives tends to diminish over time, influenced by factors such as their coping mechanisms and the support they receive from their social networks [32]. The exclusion of short timelines of sepsis-related events from the model may be because respondents did not have enough time to fully process their emotions and experiences related to these events.

#### Relationship With the Patient

The findings suggest that the relationship between the responder and the patient can have a significant impact on their emotional experience and expression of emotions related to sepsis.

The finding that responses from spouses were associated with fewer joy-related words suggests that caring for a partner with sepsis can be a challenging and stressful experience. Spouses may feel overwhelmed by the responsibilities of caregiving and the uncertainty of their partner’s health, which could contribute to a more negative emotional response overall. Additionally, spouses may also be dealing with their own emotions about losing their partner or the potential loss of their partner, further reducing the frequency of joy-related words in their responses. Studies have found that severe sepsis, in particular, can impose a significant burden on spouses, who are susceptible to the detrimental effects of psychological stress that can impair their health-related quality of life [33,34].

If the respondent was a patient who had sepsis, they were estimated to have 5.6 times more anger words as compared to responders who themselves were not patients. This could be indicative of the intense emotional experience that patients with sepsis undergo. Sepsis is a life-threatening condition that can cause physical and emotional distress. Patients who have experienced sepsis may have gone through a traumatic experience that can leave a lasting impact [23,25]. The anger expressed by the patient in their responses may be a result of their frustration with the experience of sepsis and its aftermath. They might feel angry about the loss of their independence, perceiving themselves as a burden to their loved ones who have assumed caregiving responsibilities, as well as the pain, discomfort, and disruption to their lives caused by sepsis [25]. Research has shown that sepsis survivors may experience a sense of depersonalization, feeling like they have become a different person in certain situations. This alteration in their identity can have a negative impact on their family and social relationships [23]. On the other hand, responders who were not patients may have had a more detached perspective on the situation, which could explain why they had fewer anger words in their responses. Without experiencing sepsis firsthand, they may not fully understand the emotional toll it can take on a patient.

Our findings reveal that responses provided by parents of patients with sepsis contained fewer anger- and surprise-related words. Parents may feel a sense of responsibility and obligation to remain calm and composed for the sake of their child, which could contribute to a lower frequency of anger-related words in their responses. A study conducted by Vermunt et al [35] supports this notion, as parents of children who survived septic shock reported learning to cope with the event, gaining strength from it, and developing a heightened appreciation for life.

### Guidance for Health Care Professionals

This study presents a significant contribution to the existing literature as it is the first of its kind, to the best of our knowledge, to examine the sentiments and emotions of patients and their families related to sepsis events. By focusing on this previously unexplored aspect, the study provides valuable insights into the emotional impact of sepsis on individuals and their support networks for health care professionals. Our research reveals the distinct ways in which each patient’s and their family members’ experiences with sepsis shape their overall feelings. This individuality in the experiences of patients and their families highlights the need for a comprehensive approach that includes psychological support, encourages open dialogue, involves family members, and offers educational resources. Recognizing and addressing this individuality is crucial, as it ensures that each patient and family receives personalized and holistic support tailored to their unique journey through sepsis. By addressing the emotional needs of patients and their families, health care providers can effectively alleviate fear and sadness, promote patient well-being, and enhance satisfaction with the care received.

### Limitations

Observational text analysis should be considered exploratory, as it relies on the interpretation of language patterns rather than direct measurements of emotions or experiences. As with any exploratory analysis, there are limitations to the method that should be considered. In this study, some data were missing, which meant that certain variables could not be analyzed, potentially limiting the scope of the findings. Additionally, some textual inference was completed, which means there is a possibility of error in interpreting the language patterns observed.

Responses to the blog post may originate from individuals who are not necessarily patients, are at the same or similar hospitals, of the same hospital size, or during similar times and can vary in length, among other factors that would typically result in more common responses. While this manuscript does not assert the existence of commonality among these responses, it is crucial to acknowledge that blog post data can exhibit more variability compared to other forms of electronic health record text data.

The initial story’s impact might have shaped subsequent bloggers’ narratives about their encounters, potentially biasing the spectrum of experiences shared toward those more adversely affected and potentially overshadowing milder cases in the discourse. These dynamics underscore the need for critical appraisal when interpreting these narratives to ensure a balanced understanding of the multifaceted experiences associated with sepsis events.

Further research and analysis are necessary to fully understand the relationship between patient outcomes and language use. While this study provides some insights into the language patterns associated with sepsis, additional research is needed to confirm and extend these findings. It is also important to note that lexical methods for analyzing sentiment, such as those used in this study, may not differentiate between authentic positive sentiments and sarcastic ones. Therefore, caution should be exercised when interpreting the sentiment analysis results.

### Conclusions

This study highlights the toll sepsis plays on the emotions of patients, caregivers, spouses, children, and others. This sentiment analysis of patient- and family-related sepsis events can provide valuable insights into the emotional experiences of patients and their families during these events, which can guide health care providers in providing appropriate emotional support. By acknowledging and addressing the emotional impact of sepsis, health care providers can improve patient and family experiences and outcomes.

## Appendix

Multimedia Appendix 1 Negative binomial least absolute shrinkage and selection operator regression model fit details and National Research Council of Canada emotions correlation matrix.

## Abbreviations

CDC: Centers for Disease Control and Prevention
ED: emergency department
ICU: intensive care unit
IRR: incidence rate ratio
LASSO: least absolute shrinkage and selection operator
NRC: National Research Council Canada
