# Supplementary material for: Sentiment Analysis of Patient- and Family-Related Sepsis Events: Exploratory Study
Source: JMIR Nurs. 2024 Apr 1;7:e51720. doi: 10.2196/51720 (PMC11019419; doi:10.2196/51720)
Supplement: Multimedia Appendix 1 [file nursing_v7i1e51720_app1.docx]

**Table S1**. Negative binomial Least Absolute Shrinkage and Selection Operator regression model fit details.

| **Model** | **N** | **Number of Parameters** | **BIC** | **AICc** | **Dispersion** | **Dispersion P-Value** |
| --- | --- | --- | --- | --- | --- | --- |
| Anger | 170 | 19 | 733.9 | 681.3 | 0.105 | 0.0244 |
| Anticipation | 170 | 15 | 773.6 | 731.2 | 0.178 | 0.0012 |
| Disgust | 170 | 17 | 701.4 | 653.9 | 0.126 | 0.0252 |
| Fear | 170 | 20 | 939.2 | 884.3 | 0.066 | <0.001 |
| Joy | 170 | 15 | 671.0 | 628.6 | 0.167 | 0.0059 |
| Sadness | 170 | 17 | 928.1 | 880.6 | 0.092 | <0.001 |
| Surprise | 170 | 19 | 580.8 | 528.3 | <0.001 | 0.999 |
| Trust | 170 | 16 | 842.9 | 797.9 | 0.125 | <0.001 |

^a^BIC: Bayesian information criterio.

^b^AICc: Akaike information criterion corrected.

**Table S2**. National Research Council of Canada emotions correlation matrix.

|  | **Anger** | **Anticipation** | **Disgust** | **Fear** | **Joy** | **Sadness** | **Surprise** | **Trust** |
| --- | --- | --- | --- | --- | --- | --- | --- | --- |
| Anger | 1 |  |  |  |  |  |  |  |
| Anticipation | 0.6639 | 1 |  |  |  |  |  |  |
| Disgust | 0.8478 | 0.6772 | 1 |  |  |  |  |  |
| Fear | 0.8131 | 0.804 | 0.7254 | 1 |  |  |  |  |
| Joy | 0.5755 | 0.8042 | 0.5585 | 0.6505 | 1 |  |  |  |
| Sadness | 0.8226 | 0.8129 | 0.7574 | 0.9405 | 0.709 | 1 |  |  |
| Surprise | 0.6852 | 0.7274 | 0.7236 | 0.6841 | 0.6386 | 0.6772 | 1 |  |
| Trust | 0.595 | 0.8756 | 0.5945 | 0.8045 | 0.7615 | 0.8207 | 0.6596 | 1 |
